# Efficacy of thunder-fire moxibustion in treating ankylosing spondylitis of kidney deficiency and governor meridian cold and its influence on TNF-α and RANKL: study protocol for a prospective, nonblinded, single-center, randomized controlled trial

**DOI:** 10.1186/s13063-022-06227-7

**Published:** 2022-04-23

**Authors:** Yang Liu, Pei Wang, Yan Yan Sun, Jing Qu, Min Li

**Affiliations:** Beijing Luhe Hospital Affiliated to Capital Medical University, Beijing, 101149 China

**Keywords:** Ankylosing spondylitis (AS), Thunder-fire moxibustion, Kidney deficiency and governor meridian cold, TNF-α, RANKL, Randomized controlled trial

## Abstract

**Background:**

Ankylosing spondylitis (AS) is a common chronic inflammatory spondyloarthropathy. It is considered in traditional Chinese medicine (TCM) that the pathogenesis of AS is mainly due to Yang deficiency of kidney governor meridian and internal prosperity of cold evil. Thunder-fire moxibustion is a kind of moxibustion that is characterized in abundance in drug composition, high heat radiation, and strong penetration. Thunder-fire moxibustion on the spinal segment of the governor meridian in treating AS seems compatible with the main pathogenesis of kidney deficiency and governor meridian cold. The trial aims to explore the efficacy of thunder-fire moxibustion in patients with AS of kidney deficiency and governor meridian cold and its influence on bone metabolism, through a prospective randomized trial.

**Methods:**

Sixty patients with AS of kidney deficiency and governor meridian cold will be recruited and randomly assigned to the treatment group (thunder-fire moxibustion three times a week plus basic treatment) and the control group (basic treatment) at the Center of TCM of Beijing Luhe Hospital Affiliated to Capital Medical University (Beijing, China). Each patient will be treated for 4 weeks. The primary outcome is the efficacy of TCM syndrome, and the secondary outcome indexes will include the Bath Ankylosing Spondylitis Disease Activity Index (BASDAI), Bath Ankylosing Spondylitis Functional Index (BASFI), Short-Form-36 Questionnaire (SF-36), tumor necrosis factor-α (TNF-α), and receptor activator of nuclear factor-κB ligand (RANKL). TNF-α and RANKL with observation will be determined once respectively before and after treatment, while the other indexes will be observed once prior to the treatment, 2 weeks post-treatment, and at the end of the treatment. Side effects will be recorded and analyzed as well. Inter-group comparison and analysis will be performed based on the intention-to-treat set and per-protocol set.

**Discussion:**

This prospective randomized trial will help verify the efficacy of thunder-fire moxibustion in treating AS of kidney deficiency and governor meridian cold, discuss preliminarily its mechanism in treating this disease, and provide high-quality evidences for scientific researches on clinical treatment with thunder-fire moxibustion against AS.

**Trial registration:**

Chinese Clinical Trial Registry ChiCTR2100044227. Registered on 12 March 2021

**Supplementary Information:**

The online version contains supplementary material available at 10.1186/s13063-022-06227-7.

## Administrative information

Note: The numbers in curly brackets in this protocol refer to SPIRIT checklist item numbers. The order of the items has been modified to group similar items (see http://www.equator-network.org/reporting-guidelines/spirit-2013-statement-defining-standard-protocol-itemsfor-clinical-trials/).

**Table:** 

Title {1}	Efficacy of thunder-fire moxibustion in treating ankylosing spondylitis of kidney deficiency and governor meridian cold and its influence on TNF-α and RANKL: study protocol for a prospective, nonblinded, single-center, randomized controlled trial
Trial registration {2a and 2b}.	ChiCTR2100044227 [Chinese Clinical Trial Registry], http://www.chictr.org.cn/index.aspx query=ChiCTR2100044227 [Registered on 12 March 2021]
Protocol version {3}	Version 2 of 21-01-2021
Funding {4}	Beijing Municipal funding for the development of TCM science and technology
Author details {5a}	Yang Liu1* (e-mail:865812791@qq. com),Pei Wang1 (e-mail: xiaopei349550779@126.com), YanYan Sun 1 (e-mail: zhehenhao@tom.com), Jing Qu1 (e-mail: 1027024345@qq. com),Min Li1 (e-mail: 13810048883@139.com). ^1^Beijing Luhe Hospital Affiliated to Capital Medical University, Beijing 101149, China
Name and contact information for the trial sponsor {5b}	Investigator-initiated clinical trial; Beijing TCM Administration Bureau; zyjkjc@126.com
Role of sponsor {5c}	This is an investigator-initiated clinical trial. Therefore, the funders played no role in the design of the study; collection, analysis, and interpretation of the data; and writing of the manuscript.

## Introduction

### Background and rationale{6a}

Ankylosing spondylitis (AS), a common chronic and inflammatory spondyloarthropathy, is characterized by sacroiliac joint pain and lumbodorsal morning stiffness with limited movement in the early stage. Patients with the severe disease may manifest hip joint damage, spinal ankylosis, and deformity, which are the main factors predisposing the sufferers to disabilities [1, 2]. It has been found by epidemiological researches that the incidence of AS in ordinary populations is about 0.09–0.3%, with a certain difference in various countries related possibly to the source of data or discrepancy in diagnostic criteria for this disease [3]. The incidence of AS in China is about 0.3% [4]. Due to the complexity of AS with a high misdiagnosis rate in the early stage and a high rate of disability in the advanced stage, it may induce lesions in various degrees in multiple systems or even incapacitate a sufferer, may influence the quality of life in a case of severe disease with a great psychological and economic burden on the patient, and has become gradually a serious social problem.

There is no radical cure available for AS now, and the treatment aims mainly to reduce pain, delay the progress of the disease, and improve the quality of life. In the process of treatment, patients often prefer TCM, especially the external treatment of TCM with minor side effects and great human convenience. It is of great importance, therefore, for a medical practitioner to find an effective, safe, and green therapy.

Moxibustion is a traditional treatment in TCM, using moxa or other agents for cauterizing or warmly ironing specific sites on the human body, as stated in the Yellow Emperor’s Canon of Internal that fire should be indicated for the treatment of either Yin and Yang deficiency. It is considered in TCM that moxibustion is based on meridian doctrine, and Qi got by meridians post to moxibustion may warm the body surface and is transmitted downward through the acupoints along meridians, thereby achieving the effects in warming meridians, dispersing cold, promoting Qi, activating blood circulation, and balancing Yin and Yang [5]. Its mechanism might be related to the thermal effect, radiation effect, and pharmacological function of moxibustion [6]. It is illustrated by modern medicine [7–9] that moxibustion has definite efficacy in regulating immunity, resisting inflammation, killing pain, improving bone metabolism, and maintaining bone mineral density.

A great deal of researches [10, 11] either domestic or foreign in recent years has proven that moxibustion is an important approach to treat AS, with the techniques against this disease including long snake moxibustion, indirect moxibustion, warming needle moxibustion and heat-sensitive moxibustion, all of which have thermal stimulus despite their difference in manipulating the methods and components. Thunder-fire moxibustion has the advantages such as richer components of drugs, higher temperature, greater thermal radiation, and stronger penetration [12], in comparison with ordinary moxibustions. Certain efficacy has been identified by researches [13] for thunder-fire moxibustion in treating pain and osteoarthropathy. Its indication in treating AS, therefore, is supported by evidences, despite that fewer high-quality studies have been reported on the application of thunder-fire moxibustion to the treatment of AS.

### Objectives{7}

In this research, we aim to verify the clinical efficacy of thunder-fire moxibustion in treating AS, through analyzing responses and influences on inflammation and bone metabolism in AS patients treated by thunder-fire moxibustion plus medication in comparison with those treated by medication alone, thereby discussing preliminarily the effects of thunder-fire moxibustion on arthritis and abnormal bone metabolism and providing a theoretical basis for studies on thunder-fire moxibustion applied to AS treatment and the clinical generalization for this indication.

### Trial design{8}

The study is designed as a prospective, single-center, randomized, and controlled clinical trial. Procedures in the trial lasting 6 weeks in total will include a 2-week adaption for subjects prior to randomization, followed by a 4-week treatment using a basic medication either alone or in combination with thunder-fire moxibustion.

## Methods: participants, interventions, and outcomes

### Study setting {9}

The trial will be carried out in the Center of TCM in Beijing Luhe Hospital Affiliated to Capital Medical University (Beijing China). Patients are recruited in the TCM clinic and rheumatism immune clinic of Beijing Luhe Hospital Affiliated to Capital Medical University. Patients are considered for inclusion if they meet the criteria as defined below.

### Eligibility criteria {10}

#### Primary inclusion criteria

Patients must meet the following criteria to be eligible for the study:

1. Meeting diagnostic criteria of both modern medicine and TCM
2. No exposure to any treatment within 1 month or treated only with routine drugs and without any other external treatment available
3. Aged between 18 and 70 years
4. Signing informed consent based on voluntary acceptance of treatment, with good compliance to observation and examination

#### Primary exclusion criteria

If the patients meet any of the following criteria at the screening visit, they will not be eligible for the study:

1. Aged below 18 years or above 70 years, pregnant or lactating women, or intrinsic allergy or allergic to the drug in the study
2. Complications such as severe primary diseases in the cerebrovascular system, cardiovascular system, liver, kidneys, and hemopoietic system or a patient with psychopathy
3. A sufferer of arthritis in an advanced stage, with severe deformity, rigidity, and loss of labor force

### Who will take informed consent? {26a}

Patients with AS will be screened for eligibility to participate in this study based on the abovementioned criteria. Then, they will receive initial study information. After at least 2 weeks of reflection, patients are invited to meet with the research physician to discuss any remaining questions and sign the informed consent.

### Interventions

#### Intervention description {11a}

**Fig. 1:**
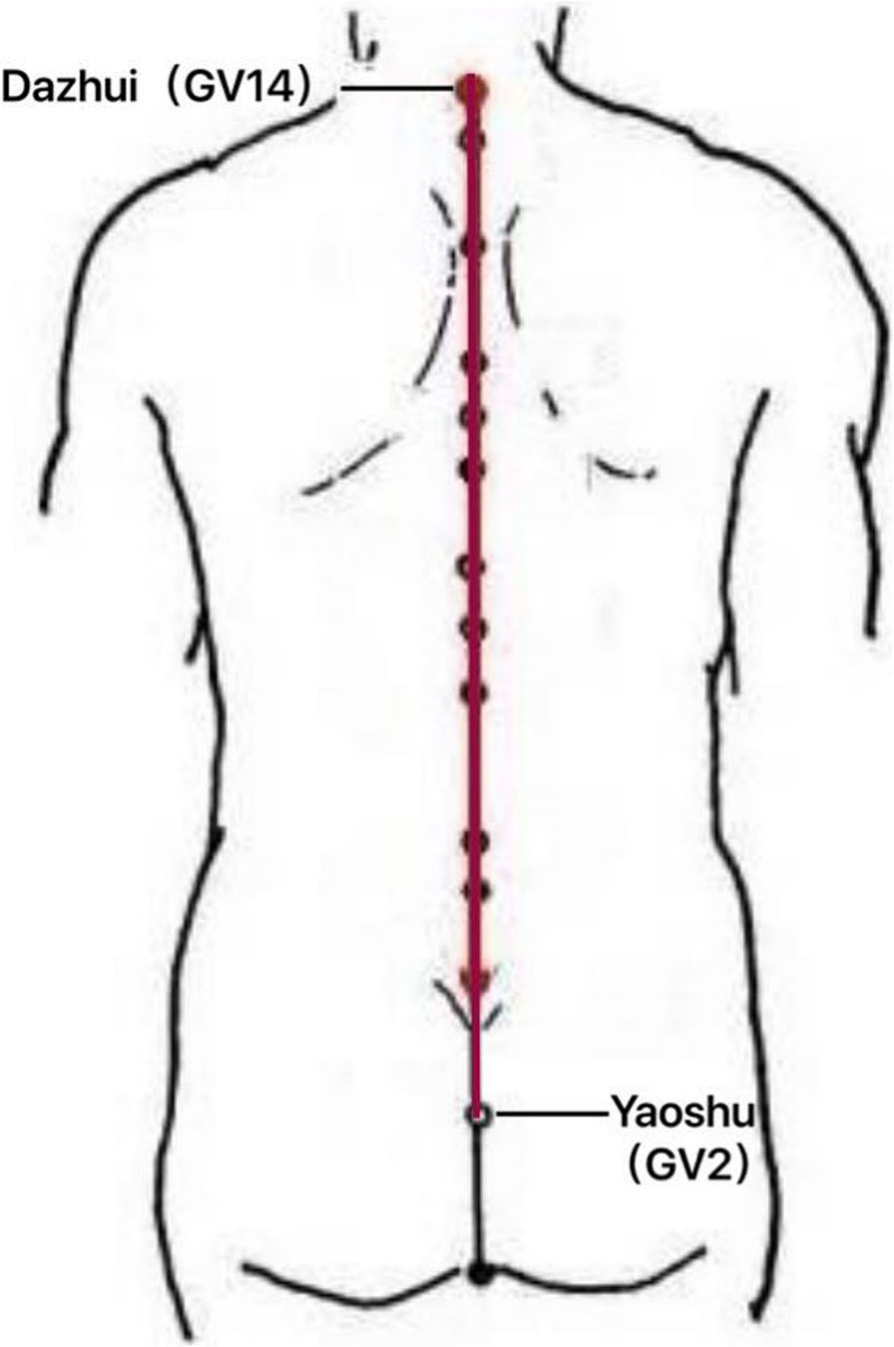
The sites for thunder-fire moxibustion. The sites will be selected along the governor meridian in its spinal segment, with the acupoints Dazhui and Yaoshu as the border

Thunder-fire moxibustion will be performed three times a week successively for 4 weeks with 12 times in total. The sites for thunder-fire moxibustion will be selected along the governor meridian in its spinal segment, with the acupoints Dazhui and Yaoshu as the borders. All acupoints will be located according to the National Standard of the People’s Republic of China: Name and Location of Acupoints (GB/T 12346-2006) issued in 2006, wherein acupoint Dazhui (GV14) is located in the infraspinous depression of the 7th cervical vertebra along the posterior middle line within the spinal region and acupoint Yaoshu (GV2) is at a place along the posterior middle line within the sacral region and opposite to sacral hiatus (see Fig. 1).

The equipment for thunder-fire moxibustion includes a thunder-fire moxa stick (28 mm × 105 mm, manufactured by Chongqing Zhao’s Thunder-Fire Moxibustion Traditional Medicine Research Institute) and the moxibustion box with 9 rear holes (provided by moxibustion room of TCM center of Beijing Luhe Hospital Affiliated to Capital Medical University).

The operational methods of thunder-fire moxibustion are as follows: the patient is kept in a prone position, and the selected acupoints are exposed. The moxibustion medicine fixed in small holes of the moxibustion box is ignited, and the box is placed onto the position of the governor meridian from acupoint Dazhui to that of Yaoshu at the back, where it is covered with a towel for a warm moxibustion for 30 min. The medicine is removed once every 15 min to blow away the ashes. The moxibustion is continued until redness appears on the skin and deep tissue becomes warm, with local perspiration. Thunder-fire moxibustion will be performed by a licensed acupuncturist with more than 6 years of experience who will be trained before the trial.

#### Explanation for the choice of comparators {6b}

The control group receives basic treatment; sulfasalazine combined with celecoxib will be used. Sulfasalazine enteric-coated tablets (manufactured by Shanghai Zhongxisunve Pharmaceutical Co., Ltd.; national medicine permission number (NMPN:H31020557)) 1.0 g b.i.d. po; in combination with celecoxib capsule (repacked by Pfizer Pharmaceuticals; NMPN: J20140072) 200 mg q.d. po. The treatment group receives thunder-fire moxibustion three times a week plus basic treatment.

#### Criteria for discontinuing or modifying allocated interventions {11b}

Participation is voluntary. Participants will be withdrawn from the study at any time for any reason. The criteria for orderly discontinuing a participant’s involvement in the study include the following: (A) the participant voluntarily withdraws from the study, (B) there are changes in their health status that make continued participation inadvisable, and (C) serious adverse events due to treatment. There will be no pre-defined criteria for modifying the allocated interventions.

#### Strategies to improve adherence to interventions {11c}

We plan to hold events to spread awareness about the treatments, which will encourage patient involvement. We will also keep in close contact with the participants if necessary throughout the study period.

#### Relevant concomitant care permitted or prohibited during the trial {11d}

During the study protocol, participants will be asked to not receive any other external therapy in TCM, and all external therapy in TCM is not permitted 1 month before the conduct of the trial.

#### Provisions for post-trial care {30}

In the event of study-related damage or injuries, Beijing Luhe Hospital Affiliated to Capital Medical University shall provide compensation, except for claims that arise from misconduct or gross negligence of involved study personnel.

### Outcomes {12}

#### Primary outcome

The primary outcome is the comparison in the efficacy of TCM syndrome between patients with AS that are treated with basic medication and patients treated with basic medication combined with thunder-fire moxibustion.

The efficacy of TCM syndrome will be evaluated by the TCM Syndrome Score (TCMSS) and TCM syndrome effective rate as the standard assessment. With the TCMSS, TCM syndrome is determined by stiffness and pain of the lumbar, sacro, ack, cervical spine and hip, fatigue, and chills (see more details in Additional file 1). The scores range from 0 to 14. Then, we will calculate the relative TCMSS by using the nimodipine method: TCMSSb stands for the TCMSS before treatment, and TCMSSa is the TCMSS after treatment: relative TCMSS = (TCMSS_b_ − TCMSSa)/TCMSS_b_ × 100%. The specific methods to assess the efficacy of TCM syndrome are as follows: (1) clinical cure: the main TCM symptoms and signs disappear, relative TCMSS ≥ 85%; (2) markedly effective: the main TCM symptoms and signs improved obviously, 65 ≤ relative TCMSS < 85%; (3) effective: the main TCM symptoms and signs improved, 30 ≤ relative TCMSS < 65%; and (4) ineffective: the main TCM symptoms and signs not improved and even aggravated, relative TCMSS < 30%. Counting the number of patients who were clinically cured and markedly effective, and denoted by *n*_1_ and *n*_2_. Calculating the TCM syndrome effective rate using the following formula: TCM syndrome effective rate = (*n*_1_ + *n*_2_)/*n* × 100%, where *n* stands for the total number of patients in this study.

#### Secondary outcomes

The Bath Ankylosing Spondylitis Disease Activity Index (BASDAI) and Bath Ankylosing Spondylitis Functional Index (BASFI) can reflect the patient’s condition more comprehensively, which will be evaluated according to the guidelines formulated by the International Ankylosing Spondylitis Evaluation Working Group in 2001 (see more details in Additional files 2 and 3, respectively). The BASDAI will be used to assess the disease activity and includes items on fatigue degree; pain or swelling degree of the neck, back, hip, and other joints; tendinitis; morning stiffness degree; and duration of morning stiffness. The scores range from 0 to 10. The BASFI will be used to determine the functional status of patients’ daily activities, which includes dressing, picking things up or taking things down, standing up from an armless seat or the floor, standing, climbing, and doing physical activities. The scores range from 0 to 10.

Short-Form-36 Questionnaire (SF-36) will be used to assess the patients’ quality of life, which is translated by the Department of Social Medicine of Zhejiang University School of Medicine in 1998 (see more details in Additional file 4). The survey includes 36 items, which are divided into 7 dimensions: physiological function (PF), bodily pain (BP), general health (GH), social functioning (SF), vitality (VT), role-emotional (RE), and mental health (MH). Convert the actual scores of each dimension into standard scores: standard score=$\frac{\mathrm{actual}\ \mathrm{score}-\mathrm{minimum}\ \mathrm{score}}{\mathrm{maximise}\ \mathrm{score}\mathrm{s}-\mathrm{minimum}\ \mathrm{score}}$× 100. The standard scores range from 0 to 100, and a higher score indicated better quality of life.

The tumor necrosis factor-α (TNF-α) is involved in inflammation and bone loss [14], and the receptor activator of nuclear factor-κB ligand (RANKL) influences the pathological processes of multiple bone metabolic diseases [15]. Thus, these two cytokines will be used to reflect the changes in bone metabolism and inflammatory condition of the patient, determined respectively prior to and post to the treatment.

#### Participant timeline {13}

**Fig. 2:**
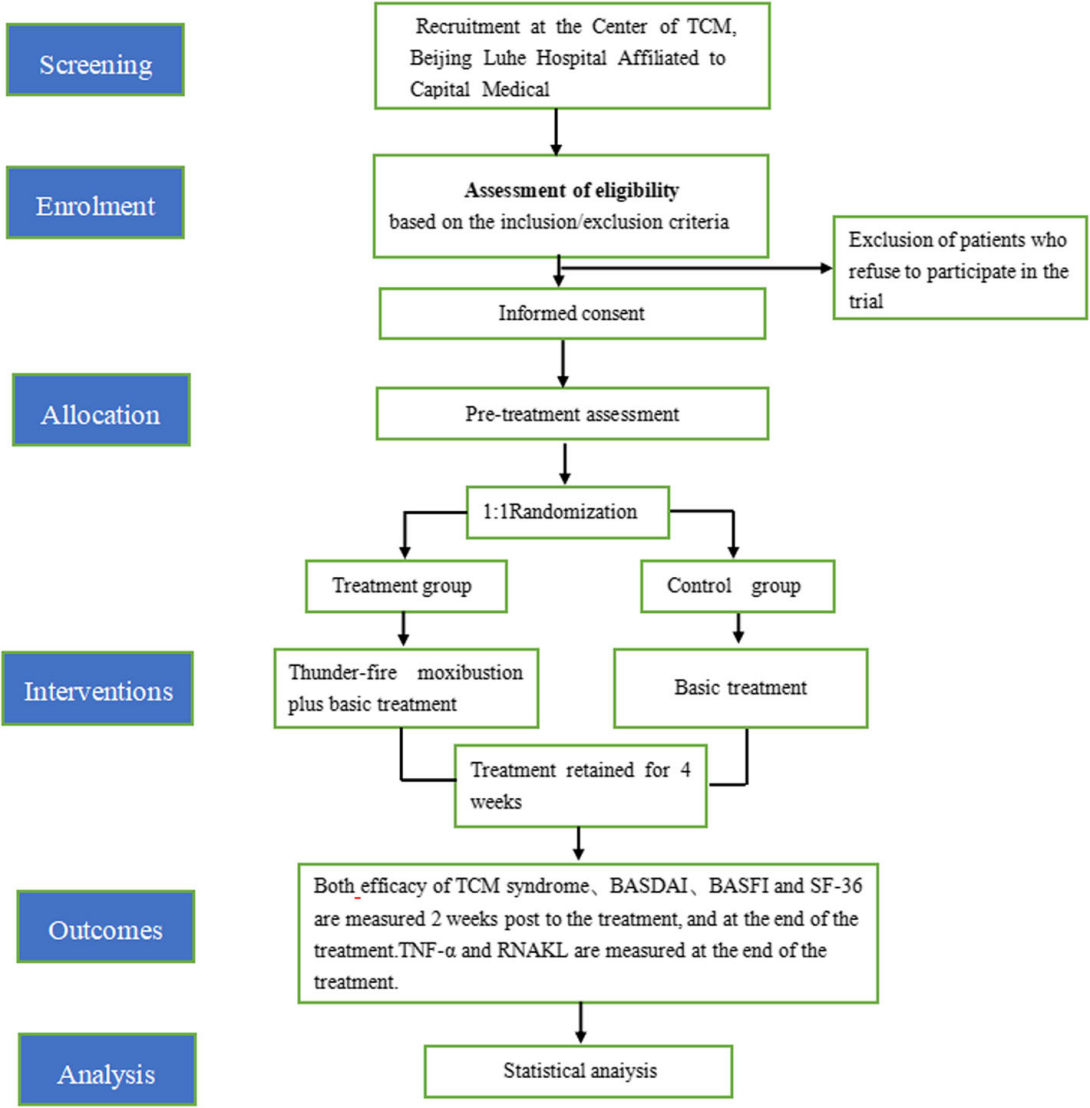
Flow chart of the trail. TCM, traditional Chinese medicine; BASDAI, Bath Ankylosing Spondylitis Disease Activity Index; BASFI, Bath Ankylosing Spondylitis Functional Index; SF-36, Short-Form-36 Questionnaire; TNF-α, tumor necrosis factor-α; RNAKL, receptor activator of nuclear factor-κB ligand

**Fig. 3:**
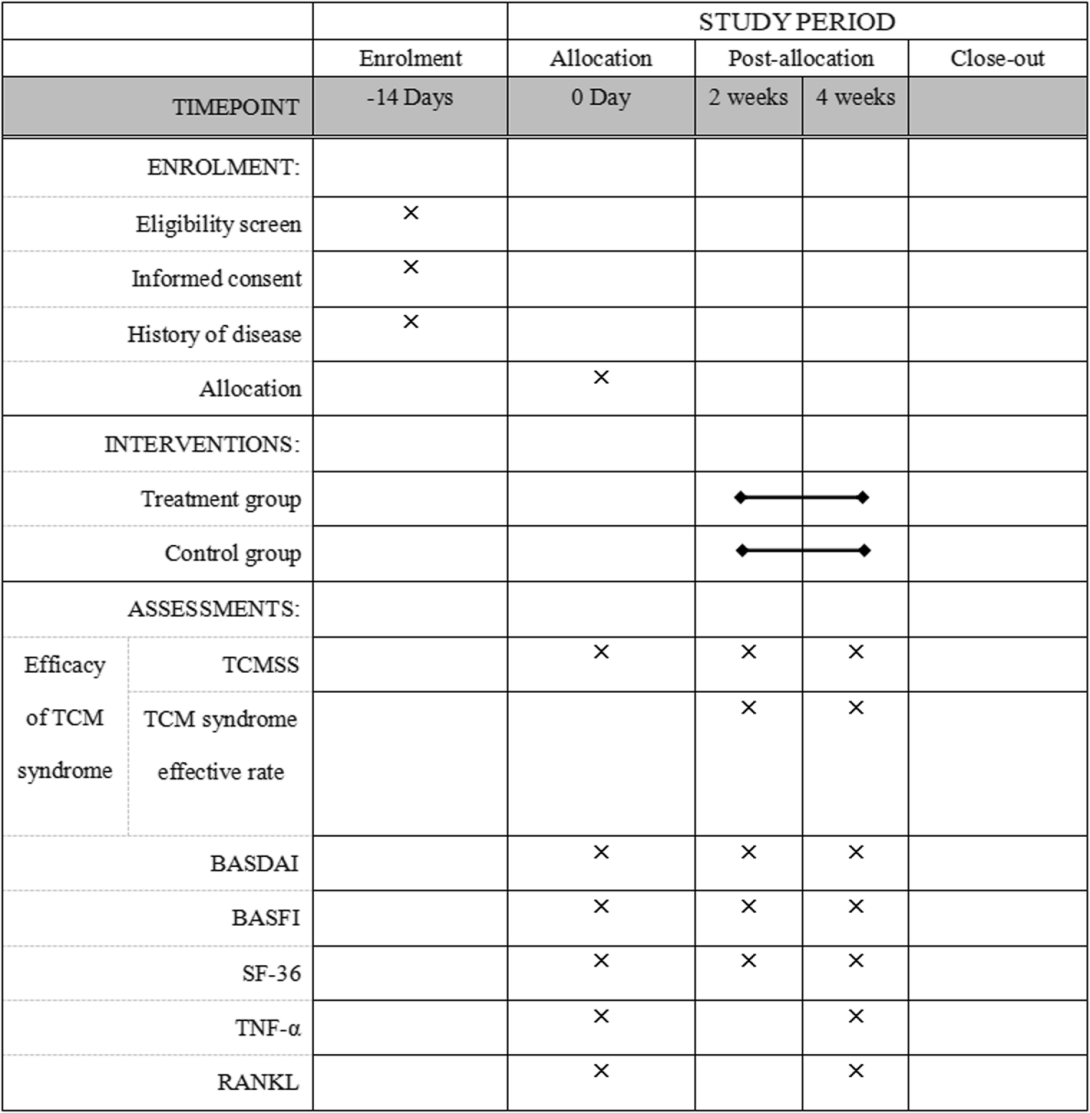
SPIRIT figure

Figures 2 and 3 show the study flowchart and the study time points, respectively.

#### Sample size {14}

Patients with AS will be randomized into two groups in a 1:1 ratio. The sample size for each group is calculated according to the following equation for a design with repeated measures [16]:

$$ {n}_c=\frac{\left[{p}_t\left(1-{p}_t\right)+{p}_c\left(1-{p}_c\right)\right]\left({\mu}_{1-\alpha /2}+{\mu}_{1-\beta}\right)}{{\left({p}_t-{p}_c\right)}^2} $$

where *n*_*c*_ stands for the number of the control group, *p*_*t*_ is the TCM syndrome effective rate of the treatment group, and *p*_*c*_ is the TCM syndrome effective rate of the control group. The values of *α*, *μ*_1−*α*/2_, and *μ*_1−*β*_ used here are as follows: *α* = 0.05 (bilateral), *μ*_1−*α*/2_ = 1.96, and *μ*_1−*β*_ = 0.84. As a result, an estimated sample size of 12 patients per group was obtained, assuming that the TCM syndrome effective rate is 97% in the treatment group and 53% in the control, based on data from clinical trials reported by the literature [17]. Fifteen subjects will be needed by each group, considering a possible drop-out rate of about 20% due to potential eliminations or drop-outs of subjects during the trial. The study may differ in manipulation methods and the selection of acupoints, in comparison with those reported in the literature, due to fewer clinical studies reported on thunder-fire moxibustion in treating AS. Therefore, the sample size is enlarged to 60 subjects in total with 30 for each group, in order to minimize the error.

#### Recruitment {15}

Patients are recruited in the TCM clinic and rheumatism immune clinic of Beijing Luhe Hospital Affiliated to Capital Medical University. Our annual clinic amount exceeds 130,000 people, which provided a guarantee for recruiting patients.

### Assignment of interventions: allocation

#### Sequence generation {16a}

Patients will be randomized into 2 parallel groups, with 30 patients in each group. The third-party personnel not participating in the study will be responsible for using the SPSS 20.0 software (SPSS Inc., Chicago, IL) to generate a random digit table. The random digits will be numbered chronologically from 1 to 60 and kept in the opaque envelops which will be also numbered sequentially from 1 to 60, then sequenced the random digits ascendingly; the numbers corresponding to the former 30 random digits sequenced will be assigned into the treatment group, while those corresponding to the latter 30 random digits sequenced will be assigned into the control.

#### Concealment mechanism {16b}

Random digits will be placed in sequentially numbered, opaque, sealed envelopes.

#### Implementation {16c}

The third-party personnel not participating in the study will use the SPSS 20.0 software (SPSS Inc., Chicago, IL) to generate a random digit table and store the envelopes with random digits. When a patient is enrolled, the envelope with its number corresponding to the sequence of his/her first visit will be opened, and the group corresponding to the random digit contained in the envelope will represent the treatment that he/she will receive.

### Assignment of interventions: blinding {17}

#### Who will be blinded {17a}

This study compared the effect of thunder-fire moxibustion therapy with conventional medicine. As it is easy to know whether thunder-fire moxibustion treatment was performed, it is unable to blind the patients and clinical practitioners. However, data collectors and statisticians were blinded in order to eliminate potential bias.

#### Procedure for unblinding if needed {17b}

The trial design is unable to blind the patients and clinical practitioners; therefore, there is no unblinding procedure.

### Data collection and management

#### Plans for assessment and collection of outcomes {18a}

Participants will complete standardized surveys prior to the treatment, 2 weeks post-treatment, and at the end of the treatment. The study staff will complete four standardized, paper-based data collection forms. These forms document the following: TCMSS based on TCM syndrome differentiation in Guiding Principles on Clinical Researches on Novel TCM Drugs in Treating Ankylosing Spondylitis (draft); BASDAI and BASFI, which will be evaluated according to the guidelines formulated by the International Ankylosing Spondylitis Evaluation Working Group in 2001; and SF-36 is translated by the Department of Social Medicine of Zhejiang University School of Medicine in 1998. Observation will be performed by a fixed researcher during the study.

TNF-α and RANKL will be determined respectively prior to and post to the treatment. Five milliliters of peripheral venous blood from the elbow will be sampled prior to and post to the treatment respectively in the morning from fasted patient and centrifuged (1800 r/min) in ultra-low temperature. The supernatant will be pipetted into an Eppendorf tube and kept in a – 80 °C refrigerator until the unified determination. Enzyme-linked immunosorbent assay (ELISA) will be used to determine the level of TNF-α and RANKL. Laboratory tests are performed by the clinical laboratory of Beijing Luhe Hospital Affiliated to Capital Medical University.

All researchers, including the acupuncturists, outcome assessors, data collectors, data managers, data entry personnel, and statisticians, will undergo training before performing the standard procedures and data management. All data will be anonymized and saved in an encryption study folder. Only the study team has access to this specific study folder.

#### Plans to promote participant retention and complete follow-up {18b}

The patients will receive initial study information and have at least 2 weeks of reflection at the start of the study. Patients are allowed to stop at any time during the study and are not obliged to give a reason to discontinue. We will distribute a 100 RMB gift to each participant at the completion of our study. Throughout the study period, the researchers will keep in close contact with the participants if necessary. If a participant chooses to discontinue the study, the data collected up to the withdrawal date will be anonymized and used.

#### Data management {19}

All data will be managed by epidata (version 3.1 http://www.epidata.dk/). After finishing all the surveys, data will be checked by trained personnel, two people both processing data to ensure data accuracy.

#### Confidentiality {27}

All participants will be anonymously enrolled in this study, and a specific alpha-numeric code will be attributed to each subject after enrolment. All paper-based data collection instruments and study-related forms will be maintained by storing them in locked filing cabinets of study investigators. The electronic data will be saved in an encryption study folder. Only the study team has access to this specific study folder.

#### Plans for collection, laboratory evaluation, and storage of biological specimens for genetic or molecular analysis in this trial/future use {33}

Five milliliters of peripheral venous blood from the elbow will be sampled prior to and post to the treatment respectively in the morning from fasted patient and centrifuged (1800 r/min) in ultra-low temperature. The supernatant will be pipetted into an Eppendorf tube and kept in a − 80 °C refrigerator until the unified determination.

### Statistical methods

#### Statistical methods for primary and secondary outcomes {20a}

All data will be analyzed using the SPSS 20.0 software (SPSS Inc., Chicago, IL). The results of continuous variables will be expressed by mean, standard deviation, and 95% confidence interval. Measurement data will be described with mean ± standard deviation (*x* ± SD), and count data will be expressed with a number of cases and percentages. All statistical analyses will use two-tailed tests, and the level of significance will be set at *p* < 0.05.

Demographic and baseline characteristics of study participants by randomization group will be analyzed by an independent-sample *t* test or nonparametric test. Data from the primary outcome (TCMSS) and secondary outcomes(BASDAI score, BASFI score, SF-36 score and TNF-α, RANKL) will be presented as continuous variables. In the comparison between the treatment group and control group, the primary outcomes and secondary outcomes will adapt the method of independent sample test or nonparametric test to analyze the efficacy of thunder-fire moxibustion in treating AS and its influence on bone metabolism. The assessment of the presence of TCM syndrome efficacy rate will be performed with the chi-square test. The relationship between treatment course and therapeutic effect in treating AS with thunder-fire moxibustion will be evaluated by the TCMSS, BASDAI score, BASFI score, and SF-36 score measured 2 weeks post-treatment and at the end of the treatment in the treatment group. They will be analyzed by the paired-samples *t*-test or nonparametric test.

#### Interim analyses {21b}

There are no interim analyses planned.

#### Methods for additional analyses (e.g., subgroup analyses) {20b}

There are no subgroup analyses planned.

#### Methods in analysis to handle protocol nonadherence and any statistical methods to handle missing data {20c}

The main comparison and analysis will be performed for outcome indexes based on the intention-to-treat set and per-protocol set.

#### Plans to give access to the full protocol, participant-level data, and statistical code {31c}

Study-related materials and study-related data would be made available upon request and with permission and approval from the Beijing Luhe Hospital Affiliated to Capital Medical University. All requests must be reasonable.

### Oversight and monitoring

#### Composition of the coordinating center and trial steering committee {5d}

The study will be monitored by an independent internal monitor. The study principal investigator and two co-investigators will be responsible for monitoring and managing data quality, assessing completeness and accuracy of data collection, implementation and adherence to the study protocol, and measurement of outcomes. Any decisions needing to be taken regarding the study will be done with the consensus of the entire study team, and the Ethics Committee of Beijing Luhe Hospital Affiliated to Capital Medical University will be notified.

#### Composition of the data monitoring committee, its role, and reporting structure {21a}

There is no data monitoring committee in this study.

#### Adverse event reporting and harms {22}

Any adverse events such as fainting, scalded, and local infection during thunder-fire moxibustion will be recorded and analyzed.

#### Frequency and plans for auditing trial conduct {23}

The Ethics Committee of Beijing Luhe Hospital Affiliated to Capital Medical University is the Trial Steering Committee and will supervise the trial, and the committee will meet once per year.

#### Plans for communicating important protocol amendments to relevant parties (e.g., trial participants, ethical committees) {25}

All protocol modifications or amendments will be reported (submitted) to the Ethics Committee of Beijing Luhe Hospital Affiliated to Capital Medical University. Participants will be notified of the amendments or modifications that impact participation, confidentiality, or safety.

#### Dissemination plans {31a}

The results of this research will be disclosed completely in international peer-reviewed journals. Both positive and negative results will be reported.

## Discussion

AS belongs to the domain of arthromyodynia, as considered by classical TCM, and is also called dyphosis or spinal arthralgia. It is stated in “Records of Tradition Chinese and Western Medicine in Combination” that “all low back pains in humans manifest the pain of the spine, which is dominated by the governor meridian…anyone with kidney deficiency must have deficiency in the governor meridian, and this is the main cause of low back pain,” indicating the close relationship between the occurrence of low back pain with kidneys and the governor meridian. This argument seems consistent with the understanding of AS pathogenesis by many contemporary medical experts [18]. It is stated in “On Generative Qi Communicating with Nature of Plain Questions” that dyphosis is generated due to invasion by cold in the case of failure in opening and closing of Yang Qi. Cold is considered to be the important cause of AS. The kidney stores essence, dominates bone, and generates marrow, so the robustness of bone correlates closely to the sufficiency of kidney essence. In addition, the kidney is of congenital origin, containing nephroyin and nephroyang of human beings and nourishing/warming human tissues and organs. The governor meridian governs Yang Qi of the whole body and is the sea of all Yang meridians. Moreover, the spine is located at the main road run through by the governor meridian and is dependent upon the prosperity and nourishment of this meridian. The bone will lose its nourishment in case of Yang deficiency in renal governor, and then the cold evil may take the advantage of weak points and invade into the body. Evils both outside and inside the body will join together, causing failure in the transformation of Yang Qi. The prevalence of cold evil goes deep into bones and joints and stays at the spine, manifesting stiffness and pain at the lumbar spine, so this disease develops. In summary, the main pathogenesis of this disease is renal deficiency with governor meridian cold.

Thunder-fire moxibustion, developed on the basis of ancient thunder-fire miraculous needle, comprises multiple drugs in specific ratios including *Artemisia argyi*, frankincense, myrrh, *Aquilaria*, Ramulus Cinnamomi, and *Notopterygium incisum* [19]. Its thermal power is strong with powerful thermal radiation while burning, capable of forming a large infrared web with a stronger penetration than that of ordinary moxibustion [20]. Medicine molecules inside the moxa stick adhere to the skin and form a region with a high concentration of drugs around the lesion, and then penetrate into deep tissues through a thermal effect. Thunder-fire moxibustion targeting the spinal segment of the governor meridian may improve local circulation of Qi and blood, eliminate cold and stop the pain, warm the meridians and facilitate Yang, and disperse the humidity and dredge meridians, which is compatible with the main pathogenesis of kidney deficiency with governor meridian cold, thereby exerting fully the advantages of thunder-fire moxibustion including powerful heat, strong penetration, and good analgesic effect and achieving the aim of direct arrival at the disease with accelerated onset of action.

From the point of view of modern medicine, the typical pathological change of AS includes inflammatory bone destruction and osteogenesis [21]. Infiltration with a great deal of inflammatory cells exists in the bones, joints, and synovial tissues in AS patients, and cytokines excreted by these cells play an important role in the chronic inflammatory process and during bone destruction and osteogenesis in AS patients [22]. As shown by researches, the proinflammatory cytokine TNF-α is involved in inflammation and bone loss [14], and a great importance attached to earlier effective anti-inflammatory treatment may relieve inflammatory bone destruction in AS patients and reduce the incidence of hyperosteogeny and arthrodesis [23]. Yu et al. [24] used conventional drugs for treating rheumatoid arthritis in combination with moxibustion, and the results showed that moxibustion could reduce the content of IL-1β and TNF-α in the serum of the patients, thereby alleviating their synovial symptoms. Similar conclusions were also obtained by the research by Luo and Liu [25]. RANKL not only regulates the biological process of bone metabolism, but also influences pathological processes of multiple bone metabolic diseases, especially the formation, survival, and activation of osteoclasts [15]. It has been proven by the results from animal experiments by Chen et al. [26] that moxibustion can lower the expression of Rankl mRNA and increase the expression of osteoclastogenesis inhibitory factor (OPG) mRNA, suggesting a recovery of balance between osteoblasts and osteoclasts, based on which moxibustion is considered capable of alleviating cartilage degradation and bone destruction in rabbit rheumatoid arthritis (RA) model possibly through modifying the OPG/RANKL signaling pathway. In summary, moxibustion may ameliorate systemic inflammation and bone metabolism disorder, and its mechanism may be related to the capability of moxibustion in regulating the content of some systemic inflammatory factors and cytokines involved in bone metabolism. Further studies are justified to explore whether the goal to ameliorate abnormal bone metabolism in AS patients can be achieved by thunder-fire moxibustion through the same route.

The study aims primarily to explore the efficacy of thunder-fire moxibustion on patients with AS of kidney deficiency and governor meridian cold and its influence on bone metabolism, enrolling AS patients as the subjects randomized into the treatment group and the control treated respectively with drugs plus thunder-fire moxibustion and with drugs alone. The clinical efficacy of thunder-fire moxibustion in AS patients with kidney deficiency with governor meridian cold will be verified based on the evaluation of indexes of clinical efficacy and related cytokines prior to and post to thunder-fire moxibustion, and the mechanism will be discussed preliminarily for this indication of thunder-fire moxibustion. The design is rigorous and capable of providing more evidences for thunder-fire moxibustion in treating AS.

## Trial status

The current protocol is version 2 of 21-1-2021. Participant recruitment began on March 20, 2020, and will end on approximately April 30, 2022.

## Supplementary Information

**Additional file 1:** TCM symptom score scale.

**Additional file 2:** BASDAI score scale.

**Additional file 3:** BASFI score scale.

**Additional file 4:** SF-36 scale.

## Abbreviations

AS: Ankylosing spondylitis
TCM: Traditional Chinese medicine
BASDAI: Bath Ankylosing Spondylitis Disease Activity Index
BASFI: Bath Ankylosing Spondylitis Functional Index
SF-36: Short-Form-36 Questionnaire
TCMSS: Traditional Chinese Medicine Syndrome Score
TNF-α: Tumor necrosis factor-α
RANKL: Receptor activator of nuclear factor-κB ligand
OPG: Osteoclastogenesis inhibitory factor
RA: Rheumatoid arthritis
