# Supplementary material for: Efficacy of thunder-fire moxibustion in treating ankylosing spondylitis of kidney deficiency and governor meridian cold and its influence on TNF-α and RANKL: study protocol for a prospective, nonblinded, single-center, randomized controlled trial
Source: Trials. 2022 Apr 23;23:344. doi: 10.1186/s13063-022-06227-7 (PMC9034605; doi:10.1186/s13063-022-06227-7)
Supplement: Supplementary file 1 — Additional file 1: TCM symptom score scale. [file 13063_2022_6227_MOESM1_ESM.docx]

**TCM symptom score scale**

| Symptom | 0 | 1 | 2 | 3 |
| --- | --- | --- | --- | --- |
| Pain in waist and hip | NO☐ | The pain is mild and does not affect work and life☐ | The pain is moderate and have effect on daily life and work☐ | The pain is severe and have a bad effect on daily life and work☐ |
| Morning stiffness duration | NO☐ | ≤30min ☐ | ＞30min and＜60min☐ | ≥60min☐ |
| Nocturnal pain | NO☐ | The pain is mild and does not affect sleep☐ | The pain is moderate and have effect on sleep☐ | The pain is severe and have a bad effect sleep☐ |
|  | NO☐ | The symptoms is mild☐ | The symptom is moderate and need to be improved by clothing☐ | The symptom is severe and could not be relieved by clothing☐ |
| Fatigue | NO☐ | YES☐ |  |  |
| No warm in the hand and foot | NO☐ | YES☐ |  |  |
| Tongue and pulse condition |  | | | |

Total score
