# Supplementary material for: Efficacy of thunder-fire moxibustion in treating ankylosing spondylitis of kidney deficiency and governor meridian cold and its influence on TNF-α and RANKL: study protocol for a prospective, nonblinded, single-center, randomized controlled trial
Source: Trials. 2022 Apr 23;23:344. doi: 10.1186/s13063-022-06227-7 (PMC9034605; doi:10.1186/s13063-022-06227-7)
Supplement: Supplementary file 2 — Additional file 2: BASDAI score scale. [file 13063_2022_6227_MOESM2_ESM.docx]

**BASDAI score scale**

| Question | Score（0-10） |
| --- | --- |
| 1.What was the overall level of fatigue / drowsiness you felt in the past week? |  |
| 2.What was the overall degree of neck, back and hip pain you've felt in the past week? |  |
| 3.What was the overall degree of other joint pain / swelling (not including neck, back and hip) you've felt in the past week? |  |
| 4.What was the overall degree of discomfort due to tenderness did you feel in the past week? |  |
| 5.What was the overall degree of morning stiffness you have felt in the past week? |  |
| 6.How long does morning stiffness persist when you are awake? |  |
| BASDAI score |  |

Instructions:

1、A score of 0 indicates no effect, and a score of 10 indicates extreme severity.

2、Duration of morning stiffness needs to be converted to VAS values before calculation：0 hour equals 0 points，1hour equals 5 points，2 hours or moreequals 10 points.

3、BASDAI score ：BASDAI=0.2×[Q1+Q 2+Q 3+Q 4+0.5×（Q 5+Q 6）].
