# Supplementary material for: Efficacy of thunder-fire moxibustion in treating ankylosing spondylitis of kidney deficiency and governor meridian cold and its influence on TNF-α and RANKL: study protocol for a prospective, nonblinded, single-center, randomized controlled trial
Source: Trials. 2022 Apr 23;23:344. doi: 10.1186/s13063-022-06227-7 (PMC9034605; doi:10.1186/s13063-022-06227-7)
Supplement: Supplementary file 3 — Additional file 3: BASFI score scale. [file 13063_2022_6227_MOESM3_ESM.docx]

**BASFI score scale**

| Question | Score（0-10） |
| --- | --- |
| 1.Wear socks or underclothes without other's help and adjunctive equipment. |  |
| 2.Bend forward and pick up pens from the floor without other's help and adjunctive equipment. |  |
| 3.Taking things from high shelves without other's help and adjunctive equipment. . |  |
| 4.Stand up from an armless seat without other's help and adjunctive equipment. |  |
| 5.Lie back on the floor first then stand up without other's help and adjunctive equipment. . |  |
| 6.Stand for 10 min without any assistive support and hold one position. |  |
| 7.Climb 12-15 steps without the banister or other aids while walk one step at a time. |  |
| 8.Not turning, looking backward from shoulder level. |  |
| 9.Do physical activities. |  |
| 10.Do a full day's work/housework. |  |
| BASFI score |  |

Instructions:

1、Patients were asked to rate how difficult it was to perform the above activities at the present time，0 is scored as easy and 10 as unlikely.

2、BASFI score：BASFI=(Q1+Q2+Q3+Q4+Q5+Q6+Q7+Q8+Q9+Q10)/10.
