# Supplementary material for: Efficacy of thunder-fire moxibustion in treating ankylosing spondylitis of kidney deficiency and governor meridian cold and its influence on TNF-α and RANKL: study protocol for a prospective, nonblinded, single-center, randomized controlled trial
Source: Trials. 2022 Apr 23;23:344. doi: 10.1186/s13063-022-06227-7 (PMC9034605; doi:10.1186/s13063-022-06227-7)
Supplement: Supplementary file 4 — Additional file 4: SF-36 scale. [file 13063_2022_6227_MOESM4_ESM.docx]

**SF-36 SCALE**

Please answer the 36 questions of the Health Survey completely, honestly, and without interruptions.

GENERAL HEALTH:

1.In general, would you say your health is:

①Excellent ②Very Good ③Good ④Fair ⑤Poor

2.Compared to one year ago, how would you rate your health in general now?

①Much better now than one year ago ②Somewhat better now than one year ago

③About the same ④Somewhat worse now than one year ago

⑤Much worse than one year ago

LIMITATIONS OF ACTIVITIES:

3.The following items are about activities you might do during a typical day. Does your health now limit you in these activities? If so, how much?

(1)Vigorous activities, such as running, lifting heavy objects, participating in strenuous sports.

①Yes, Limited a lot ②Yes, Limited a Little ③No, Not Limited at all

(2)Moderate activities, such as moving a table, pushing a vacuum cleaner, bowling, or playing golf.

①Yes, Limited a lot ②Yes, Limited a Little ③No, Not Limited at all

(3)Lifting or carrying groceries

①Yes, Limited a lot ②Yes, Limited a Little ③No, Not Limited at all

(4)Climbing several flights of stairs

①Yes, Limited a lot ②Yes, Limited a Little ③No, Not Limited at all

(5)Climbing one flight of stairs

①Yes, Limited a lot ②Yes, Limited a Little ③No, Not Limited at all

(6)Bending, kneeling, or stooping

①Yes, Limited a lot ②Yes, Limited a Little ③No, Not Limited at all

(7)Walking more than a mile

①Yes, Limited a lot ②Yes, Limited a Little ③No, Not Limited at all

(8)Walking 800 meters

①Yes, Limited a lot ②Yes, Limited a Little ③No, Not Limited at all

(9)Walking 100 meters

①Yes, Limited a lot ②Yes, Limited a Little ③No, Not Limited at all

(10)Bathing or dressing yourself

①Yes, Limited a lot ②Yes, Limited a Little ③No, Not Limited at all

PHYSICAL HEALTH PROBLEMS:

4.During the past 4 weeks, have you had any of the following problems with your work or other regular daily activities as a result of your physical health?

(1)Cut down the amount of time you spent on work or other activities

Yes No

(2)Accomplished less than you would like

Yes No

(3)Were limited in the kind of work or other activities

Yes No

(4)Had difficulty performing the work or other activities (for example, it took extra effort)

Yes No

EMOTIONAL HEALTH PROBLEMS:

5.During the past 4 weeks, have you had any of the following problems with your work or other regular daily activities as a result of any emotional problems (such as feeling depressed or anxious)?

(1)Cut down the amount of time you spent on work or other activities

Yes No

(2)Accomplished less than you would like

Yes No

(3)Didn't do work or other activities as carefully as usual

Yes No

SOCIAL ACTIVITIES:

6.Emotional problems interfered with your normal social activities with family, friends, neighbors, or groups?

①Not at all ②Slightly ③Moderately ④Severe ⑤Very Severe

PAIN:

7.How much bodily pain have you had during the past 4 weeks?

①None ②Very Mild ③Mild ④Moderate ⑤Severe ⑥Very Severe

8.During the past 4 weeks, how much did pain interfere with your normal work (including both work outside the home and housework)?

①Not at all ②Slightly ③Moderately ④Severe ⑤Very Severe ENERGY AND EMOTIONS:

9.These questions are about how you feel and how things have been with you during the last 4 weeks. For each question, please give the answer that comes closest to the way you have been feeling.

(1)Did you feel full of pep?

①All of the time ②Most of the time ③A good Bit of the Time

④Some of the time ⑤A little bit of the time ⑥None of the Time

(2)Have you been a very nervous person?

①All of the time ②Most of the time ③A good Bit of the Time

④Some of the time ⑤A little bit of the time ⑥None of the Time

(3)Have you felt so down in the dumps that nothing could cheer you up?

①All of the time ②Most of the time ③A good Bit of the Time

④Some of the time ⑤A little bit of the time ⑥None of the Time

(4)Have you felt calm and peaceful?

①All of the time ②Most of the time ③A good Bit of the Time

④Some of the time ⑤A little bit of the time ⑥None of the Time

(5)Did you have a lot of energy?

①All of the time ②Most of the time ③A good Bit of the Time

④Some of the time ⑤A little bit of the time ⑥None of the Time

(6)None of the TimeHave you felt downhearted and blue?

①All of the time ②Most of the time ③A good Bit of the Time

④Some of the time ⑤A little bit of the time ⑥None of the Time

(7)Did you feel worn out?

①All of the time ②Most of the time ③A good Bit of the Time

④Some of the time ⑤A little bit of the time ⑥None of the Time

(8)Have you been a happy person?

①All of the time ②Most of the time ③A good Bit of the Time

④Some of the time ⑤A little bit of the time ⑥None of the Time

(9)Did you feel tired?

①All of the time ②Most of the time ③A good Bit of the Time

④Some of the time ⑤A little bit of the time ⑥None of the Time

(10)During the past 4 weeks, how much of the time has your physical health or emotional problems interfered with your social activities (like visiting with friends, relatives, etc.)?

①All of the time ②Most of the time ③A good Bit of the Time

④Some of the time ⑤A little bit of the time ⑥None of the Time

GENERAL HEALTH:

How true or false is each of the following statements for you?

(1)I seem to get sick a little easier than other people

①Definitely true ②Mostly true ③Don't know

④Mostly false ⑤Definitely false

(2)I am as healthy as anybody I know

①Definitely true ②Mostly true ③Don't know

④Mostly false ⑤Definitely false

(3)I expect my health to get worse

①Definitely true ②Mostly true ③Don't know

④Mostly false ⑤Definitely false

(4)My health is excellent

①Definitely true ②Mostly true ③Don't know

④Mostly false ⑤Definitely false
